# Ownership and use of wireless telephones: a population-based study of Swedish children aged 7–14 years

**DOI:** 10.1186/1471-2458-7-105

**Published:** 2007-06-11

**Authors:** Fredrik Söderqvist, Lennart Hardell, Michael Carlberg, Kjell Hansson Mild

**Affiliations:** 1 Department of Oncology, University Hospital, Institute of Clinical Medicine Örebro University, SE-701 85 Örebro, Sweden; 2 Department of Oncology, University Hospital, SE-701 85 Örebro, Sweden; 3 Department of Oncology, University Hospital, SE-701 85 Örebro, Sweden; 4 Department of Radiation Physics, Umeå university, SE-90187 Umeå, Sweden

## Abstract

**Background:**

Recent years have seen a rapid increase in the use of mobile phones and other sources of microwave radiation, raising concerns about possible adverse health effects. As children have longer expected lifetime exposures to microwaves from these devices than adults, who started to use them later in life, they are a group of special interest.

**Methods:**

We performed a population-based study to assess ownership and use of mobile phones and cordless phones among children aged 7–14 years. A questionnaire comprising 24 questions was sent to 2000 persons selected from the Swedish population registry using a stratified sampling scheme.

**Results:**

The response rate was 71.2%. Overall, 79.1% of the respondents reported mobile phone access, and 26.7% of them talked for 2 minutes or more per day. Of those who reported mobile phone access, only 5.9% reported use of hands-free equipment. Use of cordless phones was reported by 83.8% of the respondents and 38.5% of them talked for 5 minutes or more per day. Girls generally reported more frequent use than boys.

**Conclusion:**

This study showed that most children had access to and used mobile and cordless phones early in life and that there was a rapid increase in use with age. It also showed very low use of hands-free equipment among children with mobile phone access, and finally that girls talked significantly more minutes per day using mobile and cordless phones than boys did.

## Background

Over the last years there has been a rapid worldwide increase in the use of mobile phones and other sources of microwave radiation. According to an industry research institute based in Taipei, the global number of mobile phone subscribers hit 2.3 billion in 2006, and this is expected to climb to 3.3 billion by 2011 [1]. Many European countries now have a mobile phone penetration rate of more than 100%, which is to say there are more subscribers than inhabitants [2]. In December 2005 Sweden had a penetration rate of 112% and during 2006 3.2 million mobile phones were sold on the Swedish market, a 15% increase over the previous year [2,3].

The potential long-term health consequences of these developments can be difficult to predict and may therefore give rise to considerable public concern. Most epidemiological studies on radiation from hand-held mobile phones and possible adverse health effects have shown no increased risk [4], but whether the existing studies have had sufficiently long follow-up times to permit firm conclusions regarding chronic diseases is still debatable [5,6].

Furthermore, potential endpoints more relevant to mobile phone use by children have been suggested [7,8], such as the impact of electromagnetic fields on the child's developmental process. Unfortunately, the number of biological studies relevant to this field is limited [4,9], which is one of the reasons why the WHO research agenda [10] from 2006 emphasizes the need for studies on children and radio frequency exposures. This agenda states that research is needed to document the rapidly-changing patterns of wireless communication use because such a large proportion of the population is exposed.

To date, only two systematic studies have been conducted and published in which the primary aim was to survey ownership and use of mobile phones among children [11,12]. We therefore conducted a population-based study with the primary aim of assessing ownership and use of mobile phones and cordless desktop phones (DECT) by Swedish children and adolescents aged 7–14 years. The second aim of the study was to examine factors that could explain mobile phone access and regular mobile phone use in these two groups.

## Methods

The study methods were checked and approved by the local ethics committee. A stratified sampling scheme was used to recruit 2000 individuals, so 125 girls and 125 boys were randomly selected for each year group in the 7–14 range from the Swedish Population Registry. We then used the population registry, which contains information about current municipality for all residents such as geographical location, and links the subject's living area code to a so-called homogeneity region, classified by Statistics Sweden [13]. Six different regions are categorized by population density and the number of inhabitants in the vicinity of the main city in that municipality. The two highest density categories (H1, H2) are represented by the largest cities of Sweden, Stockholm, Göteborg and Malmö/Lund. H3 consists of municipalities with more than 90,000 inhabitants within a 30 km radius from the centre. H4 includes municipalities with more than 27,000 but fewer than 90,000 inhabitants within a 30 km radius from the centre, but also more than 300,000 inhabitants within a 100 km radius from the same centre. H5 is the same as H4, except that it has fewer than 300,000 inhabitants within the same radius. H6 includes municipalities with fewer than 27, 000 inhabitants within a radius of 30 km from the centre.

For data collection, a specially designed questionnaire along with a letter of information was sent to each study subject's guardian. The letter stressed the importance of cooperation between the child and the guardian in answering the questionnaire. All questionnaires (n = 2000) were mailed to the study subjects during October 2005 and returned before the end of May 2006 (n = 1423). Two reminders, and supplementary questions if necessary, were sent by mail to improve data quality. Those who had not answered the questionnaire after two reminders were regarded as non-responders.

The questionnaire comprised 24 questions and the respondents had either to tick the appropriate alternative or to write down some numbers, and if necessary also comment on them. The first 9 questions concerned background data such as sex, age, number of siblings, height, weight, type of school, age of parents or guardians and income of household. The following questions concerned placement and interaction with various wireless devices such as mobile phones and DECT, wireless Internet connection at home or in school, and wireless earphones and wireless music equipment.

Finally, questions were asked about TV-watching, sleep habits and physical activity. Respondents were asked about twenty different types of physical activity and their duration in hours per week; 1–7, 8–14 or > 14 hours. An open question was also included in case the listed activities did not apply to the respondents or any activities were missing. Information from these questions was then used for analyses of factors that could explain mobile phone access and regular mobile phone use. The distribution of the study base was classified on the basis of these two outcome variables. Mobile phone access was defined as having access to one's own or another mobile phone, e.g. one shared within the family. Regular mobile phone use was defined as talk for ≥ 2 min per day and regular DECT use as talk for ≥ 5 min per day.

First frequency tables were produced for background variables as well as those showing use of wireless technology equipment and other health-related issues of interest. Some specific questions relating to the aim of the study were then chosen for further analysis to determine any differences between, for instance, age and gender in respect to mobile phone or DECT use. Questions concerning differences between groups in use of wireless devices were examined by χ^2^. Unconditional logistic regression analyses were performed to calculate the odds ratios (OR) and 95% confidence intervals (CI) for factors explaining access to and regular use of mobile phones, where each explanatory factor was separately adjusted for age, sex and family income; these variables were significantly associated with regular mobile phone use according to the χ^2 ^test. Dependent variables were mobile phone access/no mobile phone access and regular mobile phone use/no regular mobile phone use. Independent variables were explanatory factors such as H-regions, siblings yes or no, overweight condition and obesity, time spent watching TV, time spent playing computer games, physical activity, use of DECT, hours of sleep and sufficiency of sleep. Overweight condition and obesity were defined as suggested by Cole et al. [14]. Physical activity was classified into three groups according to the number of hours per week. We adjusted for age as a continuous variable and for household income using three categories (see Table 1) with the average income group as reference (OR = 1.0). Household income in euros per year was defined as: below average < 21,100, average ≥ 21,100 – 47,500 and above average > 47,500. Stata 8.2 was used for all statistical analyses (Stata/SE 8.2 for Windows; StataCorp, College Station TX).

**Table 1:** Reported mobile phone access and regular mobile phone use in different age groups and according to sex and household income

	Children who reported mobile phone access			Children who reported regular use *		
	Total in category	%	N	Total in category	%	N

Age (in years)						
7	165	49.1	(81)	76	7.9	(6)
8	184	47.3	(87)	85	9.4	(8)
9	158	75.3	(119)	109	12.8	(14)
10	186	80.6	(150)	145	19.3	(28)
11	192	90.1	(173)	172	14.5	(25)
12	182	92.3	(168)	165	34.5	(57)
13	175	97.1	(170)	167	44.9	(75)
14	179	98.3	(176)	176	44.9	(79)
p, χ2-test		<0.001			<0.001	

Sex						
Female	712	81.2	(578)	566	31.8	(180)
Male	709	77.0	(546)	529	21.2	(112)
p, χ2-test		0.053			<0.001	

Household income						
<Average	170	73.5	(125)	122	36.1	(44)
Average	700	79.0	(553)	538	26.6	(143)
>Average	514	81.7	(420)	410	23.7	(97)
p, χ2-test		0.07			0.02	

* = Defined as talking for ≥ 2 min per day among those who reported mobile phone access.

## Results

After two reminders, 1423 (71.2%) individuals answered the questionnaire. Among the respondents, 49.8% were boys and 50.2% girls. There was no age-related trend in differences in response rate and there were only marginal differences over the different population density regions. One gender difference was seen in H6 (43.2% boys and 56.8% girls) but this group was quite small (n = 74 of the total 1423) and no differences were seen in other regions. The percentage of missing data for separate questions was greatest for height (4.8%), weight (6.4%) and age of father (3.6%). For most questions, however, missing data did not exceed one percent.

**Figure 1:**
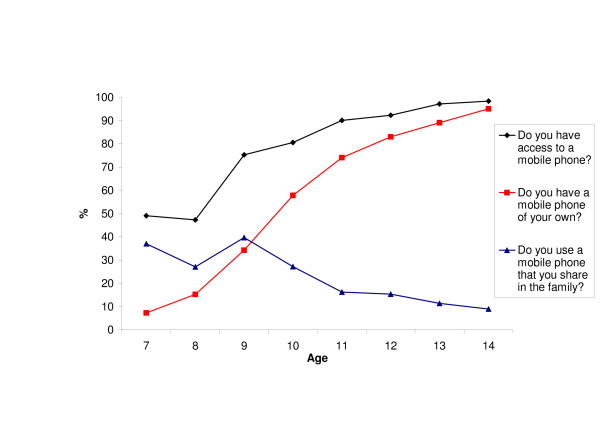
Percentage of children aged 7–14 years with access to a mobile phone, own mobile phone or shared in the family.

Among all respondents, 79.1% reported access to a mobile phone and 57.7% reported a mobile phone of their own. The most commonly-used type of mobile phone, reported by 68.9% (n = 971) of all respondents, was the digital GSM phone, while 9.7% (n = 138) reported owning a 3G phone and 0.6% (n = 9) an analogue phone (Nordic Mobile Telephone System; NMT). Among children aged 7 years, 49.1% had access to a mobile phone. Ownership was reported by 7.3% (n = 12) in that age group, a percentage that increased rapidly with age to 57.8% among 10-year-olds and 95.0% in the 14-year age group (Figure 1).

**Figure 2:**
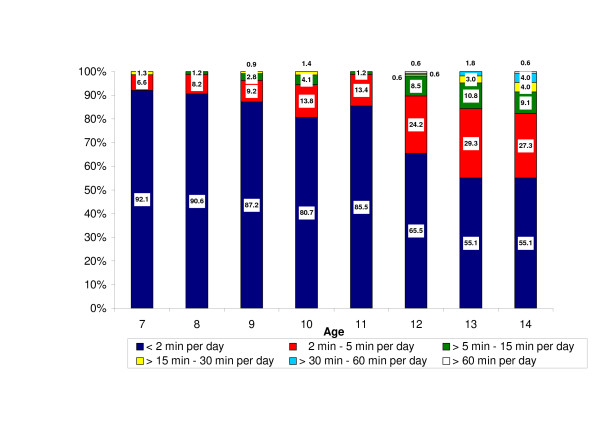
Percent distributions of average use in minutes per day of own mobile phone in the different age groups.

Across all age groups, 73.3% (n = 803) reported talking by mobile phone < 2 min per day, 18.4% (n = 202) 2–5 min per day, 5.5% (n = 60) > 5–15 min, 1.6% (n = 17) > 15–30 min per day and 1.2% (n = 15) > 30 min per day. Mobile phone use across the different age groups is summarized in Figure 2. Children aged 14 years reported the most frequent use. Figure 2 also shows that there was a steep increase in regular users (who talked for ≥ 2 min per day) with age, from 7.9% (n = 6) of the 7-year-old children to 44.9% (n = 79) in the 14-year age group. Overall, 26.7% (n = 292) were regular users.

There were significant gender differences in mobile phone use. Of the girls, 68.2% (n = 386) reported talking for < 2 min per day compared with 78.8% (n = 417) of the boys; 20.7% (n = 117) compared with 16.1% (n = 85) talked for 2–5 min per day, 7.4% (n = 42) compared with 3.4% (n = 18) for 5–15 min per day, 2.3% (n = 13) compared with 0.8% (n = 4) for 15–30 min per day, 1.2% (n = 7) compared with 0.8% (n = 4) for 30–60 min per day, and 0.2% (n = 1) compared with 0.2% (n = 1) >60 min per day. Mobile phone access was reported by 81.2% of the girls compared with 77.0% of the boys (P = 0.053) and regular use by 31.8% of the girls compared with 21.2% of the boys (P < 0.001); see Table 1.

The reported average use of hands-free equipment varied with age. Overall, 5.9% (n = 66) of those with access to a mobile phone used hands-free. The 14-year-olds reported the highest average use, 11.4%. Wireless hands-free equipment was reported by 2.5% of the respondents. Sending short text messages (SMS) was common among participants who reported mobile phone access; 65.3% used their phones to send and receive SMS. Girls also reported sending more SMS than boys; 70.2% (n = 406) compared with 60.1% (n = 328) (P < 0.001).

**Figure 3:**
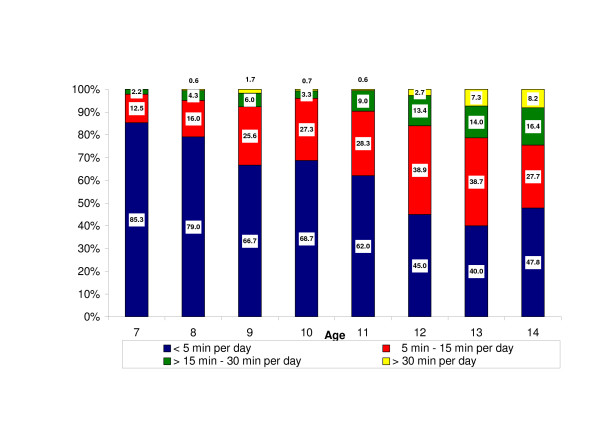
Percent distributions of average use in minutes per day of cordless phone in the different age groups.

Regarding type of home telephone, 83.8% of the respondents reported having a DECT. Of these, 36.6% had only a DECT, 47.2% had both a DECT and a regular landline phone, and 11.5% reported only having a landline phone. No home phone was reported by 4.4%, and 0.2% did not define type of phone. Figure 3 describes the use of DECT per day by the different age groups. The average use increased with age as for use of mobile phones, but clearly there were more regular users of DECT (= 5 min per day) than of mobile phones.

Gender differences were also seen in DECT use. Among participants who reported having a DECT at home, 52.8% (n = 316) of the girls compared with 70.2% (n = 415) of the boys talked for < 5 min per day, 31.3% (n = 187) compared with 22.7% (n = 134) for 5–15 min per day, 11.7% (n = 70) compared with 5.8% (n = 34) for 15–30 min per day, and 4.2% (n = 25) compared with 1.4% (n = 8) reported talking for > 30 min per day. When we combined reported access to mobile phone and DECT, we found that 95.0% of the children had access to either one or both phone types and there were no gender differences in this respect.

Use of a wireless Internet connection in school was reported by 15.3% of the participants while 22.1% reported use during their leisure time. Use of a wireless computer mouse was reported by 27.1% and a wireless keyboard by 19.6%. Use of wireless stereo or home cinema equipment was reported by 3.4%, wireless music headphones by 2.3% and walkie-talkie by 8.6%. Gender differences were marginal but girls reported slightly more use overall, except for walkie-talkie. Use of these devices was also, with the exception of walkie-talkie, less age-related than use of mobile phone and DECT. Walkie-talkie was more commonly used by children aged 7–11 years than by 12-14-year-olds (12.1% compared with 2.8%).

Table 1 shows that mobile phone access and regular use increased with age so that almost all (98.3%) children aged 14 years had access to mobile phone and 44.9% used it regularly. Both access and use were more common among girls than boys. Access to a mobile phone was most common among children in families with more than average income. The opposite was found for regular use of a mobile phone, which was most common among children in families with below-average household income. Differences between the groups were significant (P = 0.02).

Table 2 shows the factors that may explain mobile phone access and use. Significant associations with regular use were found for time spent watching TV and use of DECT. Sleep < 7 hours yielded OR = 3.6, 95% CI = 1.1–12, and not enough sleep gave OR = 1.5, 95% CI = 1.1–2.2 for regular mobile phone use. However, mobile phone access was not associated with lack of sleep. It seemed to be more common in more sparsely populated areas, but regular use was less frequent in such areas. If we combined H4-H6 and compared them with H1-H3, this yielded OR = 1.5, 95% CI = 1.1–2.1 for mobile phone access and OR = 0.6. 95% CI = 0.5–0.9 for regular use among children living in H4-H6 (data not in Table). Having siblings gave decreased OR for both access and use of mobile phone.

**Table 2:** Odds ratio (OR) and 95% confidence interval (CI) for factors that might explain mobile phone access and regular use. Unconditional logistic regression analysis adjusted for age, gender and household income was used. Number of "exposed" (access or regular use) and "unexposed" (no access, no regular use) in the different categories is shown.

	Children who reported mobile phone access			Children who reported regular use*		
	Access/no access	OR	95 % CI	Regular use/no regular use	OR	95 % CI

Household income**						
Average	553/147	1.0	-	143/395	1.0	-
<Average	125/45	0.6	0.4 – 0.9	44/78	1.5	0.98 – 2.4
>Average	420/94	1.3	0.96 – 1.8	97/313	0.9	0.7 – 1.3

H-regions						
H1	200/54	1.0	-	69/128	1.0	-
H2	163/47	1.0	0.6 – 1.7	53/104	0.8	0.5 – 1.4
H3	390/122	0.9	0.6 – 1.3	89/291	0.5	0.3 – 0.7
H4	224/45	1.3	0.8 – 2.2	41/177	0.3	0.2 – 0.5
H5	83/19	1.2	0.6 – 2.3	26/55	0.8	0.4 – 1.5
H6	64/10	2.0	0.9 – 4.6	14/48	0.5	0.2 – 0.9

Siblings						
No	83/14	1.0	-	29/53	1.0	-
Yes	1041/283	0.4	0.2 – 0.8	263/750	0.5	0.3 – 0.8

Overweight						
No	888/223	1.0	-	236/633	1.0	-
Yes	156/43	1.1	0.7 – 1.7	35/115	0.9	0.6 – 1.4

Obesity						
No	1019/254	1.0	-	268/727	1.0	-
Yes	25/12	0.6	0.3 – 1.3	3/21	0.4	0.1 – 1.4

Spent time watching TV						
< 30 min per day	70/30	1.0	-	10/57	1.0	-
≥ 30 – 60 min per day	347/132	1.2	0.7 – 2.0	78/260	2.0	0.9 – 4.2
> 60 – 180 min per day	601/124	1.8	1.05 – 3.2	157/429	2.2	1.1 – 4.6
> 180 min per day	97/10	2.8	1.1 – 6.9	45/51	3.6	1.6 – 8.4

Spent time playing computer games						
Never	143/34	1.0	-	65/75	1.0	-
< 30 min per day	422/137	1.5	0.9 – 2.5	97/313	0.6	0.4 – 0.997
≥ 30 – 60 min per day	298/84	1.7	0.98 – 3.0	63/231	0.6	0.4 – 1.03
> 60 – 180 min per day	196/37	1.8	0.97 – 3.5	53/134	0.7	0.4 – 1.3
> 180 min per day	55/1	8.0	0.999 – 64	12/42	0.4	0.2 – 0.96

Regular physical activity						
1–7 hours per week	792/230	1.0	-	188/582	1.0	-
8–14 hours per week	146/15	1.6	0.8 – 3.0	46/99	1.2	0.8 – 1.9
>14 hours per week	10/4	0.2	0.1 – 0.9	6/4	4.7	1.1 – 19

Use of DECT						
Never	136/64	1.0	-	38/92	1.0	-
< 5 min per day	541/190	1.7	1.1 – 2.5	74/446	0.4	0.3 – 0.7
≥ 5 – 15 min per day	295/26	4.3	2.5 – 7.4	91/202	1.0	0.6 – 1.6
> 15 – 30 min per day	97/6	5.2	1.9 – 14	58/39	2.8	1.5 – 5.0
> 30 min per day	32/1	4.3	0.5 – 35	26/6	5.7	2.1 – 16

Hours of sleep						
7–9 hours	735/119	1.0	-	230/491	1.0	-
< 7 hours	15/2	0.5	0.1 – 3.0	10/5	3.6	1.1 – 12
> 9 hours	371/175	0.8	0.6 – 1.1	51/305	0.6	0.4 – 0.8

Enough sleep						
Yes	922/262	1.0	-	211/684	1.0	-
No	185/31	0.9	0.6 – 1.5	75/108	1.5	1.1 – 2.2

* = Defined as talking for ≥ 2 min per day among those who reported mobile phone access.

** = Adjusted for age and gender.

Household income did not significantly affect mobile phone use. Above-average income gave somewhat increased OR = 1.3, 95% CI = 0.96–1.8 for mobile phone access and OR = 0.9, 95% CI = 0.7–1.3 for regular use. Increased OR for mobile phone access was found among children playing computer games, but this was not associated with regular use; > 180 min per day gave OR = 0.4, 95% CI = 0.2–0.96. For children with physical activity > 14 hours/week we obtained OR = 0.2, 95% CI = 0.1–0.9 for mobile phone access and OR = 4.7, 95% CI = 1.1–19 for regular use, but these results are based on low numbers.

## Discussion

The lack of knowledge regarding the use of wireless telephones among children, and recent trends that seem to show increasing frequency of mobile phone use, emphasize the need for more research on potential long-term health effects and the close monitoring of user habits. The main aim of this population-based study was to assess ownership and use of mobile phones and DECT. The results showed that mobile phone use started early in life and, as expected, the GSM phone was the most commonly-owned and used type. In total, 95.0% of the children reported access to either a mobile phone or a DECT, or both.

The results also showed a rapid increase in the number of regular users with age. Of those who reported access to a mobile phone among the 14-year-olds, 44.9% (n = 79) were regular users; they talked for 2 min or more per day. Overall, 26.7% (n = 292) of the children were regular users. The reported use of hands-free equipment varied across age groups but was generally very low; 14-year-olds reported the most frequent use.

We found age and gender differences: both mobile phone and DECT use increased with age and was more common among girls (Table 1). Girls also sent more SMS than boys did. Almost half the children aged 7–8 years reported access to a mobile phone, but fewer than 10% of those were regular users. Mobile phone access increased with age and almost all children aged 14 years (98.3%) had access, though as mentioned only 44.9% of those were regular users. This probably indicates that among the younger children, mobile phones were mainly used for purposes other than talking just for fun, such as expedient contact with parents in an emergency.

In Table 2, the unconditional logistic regression analysis adjusted for age, sex and household income revealed further statistically significant factors, which predicted mobile phone access and regular use. When we compared the factors relevant to access with regular use, some results differed. The most sparsely populated region (H6) gave OR = 2.0, 95% CI = 0.9–4.6 for mobile phone access, compared with OR = 0.5, 95% CI = 0.2–0.9 for regular use. There is no obvious explanation for these results, but perhaps parents' desire to be able to contact their children is greater in sparsely populated areas, for instance because distances between home and school are long. Increasing time spent playing computer games increased the OR for mobile phone access but not for regular use, which may be due to social factors. Insufficient sleep was not associated with access but gave increased OR for regular use. Some of these results, however, were based on low numbers.

Factors that predicted regular mobile phone use were watching TV, use of DECT, regular physical activity, sleeping less than 7 hours and insufficient sleep. Thus, these variables seemed to be correlated with each other as life-style factors. In contrast, living in a sparsely populated area, having siblings and playing computer games were factors that predicted less mobile phone use. As shown in Table 2, children whose guardians reported below-average income were less likely to have access to a mobile phone, but those in this group who did report access tended to use their mobile phones more regularly than those who reported average income. This finding was based on low numbers but similar results were obtained in a Finnish study that looked at reported mobile phone use among adolescents in relation to guardians' education [15]. We also found in our study that children whose guardians reported above-average income tended to report more mobile phone access and ownership (data not shown) than those who reported average income. The reasons for these findings – if they are real and not due to chance – are unclear, but one hypothesis could be that it is easier for parents with above-average incomes to gain access to and provide their child with a mobile phone, while they are also more aware of potential health risks and therefore exert more control over the child's use. On the other hand, the latter is contradicted by that fact that so few used hands-free equipment.

Some of our results differ from those of the German study [12], one of the two existing similar studies [11,12], in that the proportions of mobile phone owners and users were higher for socially disadvantaged children. However, we used below-average income as a marker of socioeconomic status while in the German study this was based on a schoolteacher's judgement [12], so the results might not be comparable. In contrast to the German study, our study and the Hungarian one [11] also found that the boys were less likely than girls to be regular mobile phone users.

There was some similarity in the results regarding factors that could explain regular mobile phone use, e.g. time spent watching TV, physical activity and sleep habits. However, our study is only partially comparable with the other two because of differences in design. For example, the German and Hungarian studies used the same questionnaire and surveyed mostly 9-11-year-old children, from primary schools in one city in Germany and three cities in Hungary. Our study was population-based and included a sample of children from the whole of Sweden.

The aim of this study was not to explore explanatory factors in detail. For that, in-depth interviews would be necessary. For example, regular physical activity of more than 14 hours/week was significantly associated with regular mobile phone use. However, because regular physical activity was defined by number of hours/week regardless of type of physical activity and the analysis was based on low numbers, this finding must be interpreted with caution.

There are several limitations to our study that might have affected the results. One potential bias could be a sex difference in the 7–14 year age group in Sweden, since equal numbers of boys and girls were drawn from the Population Registry. In 2005 there were 51.3% boys and 48.7% girls according to the Population Registry [16], compared to 49.8% boys and 50.2% girls among the respondents in our study. However, this difference is not statistically significant (P = 0.28). At the end of 2005, the number of children in Sweden was unequally distributed among the 7–14 year age groups: there were somewhat more children in the older than in the younger groups. Compared with the distribution among the respondents in our study, the difference was significant (P = 0.01). Since use of wireless phones increased with age, the overall results may therefore be underestimates. However, we also present age-specific results that are not biased in this respect.

The participation rate was 71.2%, so we cannot exclude the possibility that response bias might have influenced some of the findings. Therefore we compared reported mobile phone access and use by early responders with responders who were sent at least one reminder. While there was no difference with regard to access (P = 0.95), there was difference for use (P = 0.01): the responders who were sent at least one reminder used their mobile phone more than early responders. This could also mean that reported use was underestimated in this study.

We also saw differences between girls and boys in use of both mobile phone and DECT. If this was due to bias, then the girls either over-reported their use, and/or regular users among the boys under-reported their use or did not answer the questionnaire. However, there were no differences between girls and boys either in response rate or in the distinction between early responders and those who were sent at least one reminder (P = 0.94).

Another possible source of response bias is that several questions might have been difficult for young children to answer. We did address the letter of information to the guardian and stressed the importance of assisting the child in answering the questionnaire. Unfortunately, no question was included to show whether the child and the guardian together, or only one of them, had answered the questionnaire. This could also have altered the results. However, if the questions were too complicated for the younger children to answer, then we would expect there to be an age difference between early and late responders, so that more young children would only have answered after at least one reminder. We could see no such significant difference (P = 0.18).

Although we did assess use of hands-free equipment, we could also have asked questions regarding other means of reducing unnecessary exposure such as talking only when the mobile phone has a good communication with the base station. Another weakness of this study concerns the durability of the data. Since this was a prevalence study it only gave information about average mobile phone use (for example) during a limited time period. It did not cover changes in use over a longer time period. However, other findings of this study such as the differences between girls and boys in both mobile phone and DECT use are not as likely to be time-related.

Finally, some comments should be made about the likely validity and reliability of the estimates of wireless phone use in this study, and also about our choice of method. Other sources of information on mobile phone and DECT use would have been valuable for comparison. However, no such information about DECT use was available among the telephone operators, since no information on the type of telephone used was registered. Similar problems are related to mobile phone use, since use of pay-as-you-call cards is likely to be common. Several children do not have their own mobile phone, especially the younger ones, and billing records give no information about incoming calls. Contacts with telephone operators have shown that it is not easy to obtain information about mobile phone use over time owing to the structure of their data. It is therefore difficult to say how reliable the estimates of use in this study are.

Ideally, in future studies, comparing the estimates with the true cumulative times of incoming and outgoing calls should test their reliability. This can be done using specially software-modified phones handed out to a sample of the respondents. Preferably these phones should not only record the number of calls but also the output power during operation. Such devices exist and have been used e.g. for validating self-reported mobile phone use in the Interphone Study [17]. Regarding validity, we checked for possible bias in the analysis and found that some results could have been somewhat underestimated, e.g. overall use of mobile phones. A strength of this study, however, was its population-based design which, if not flawed with systematic errors, makes the results representative of all Swedish children aged 7–14 years.

## Conclusion

In conclusion, this study showed that most Swedish children in the 7–14 age group seemed to begin mobile phone and DECT use early in life and that there was a steep increase in use with age. It also showed that girls generally talked for significantly more minutes per day using mobile phones and DECT than boys, and that the frequency of use of hands-free equipment was very low among children with mobile phone access.

## List of abbreviations

Digitally enhanced cordless phone (DECT)

Global system for mobile communication (GSM)

Homogeneity regions (H)

Nordic Mobile Telephone System (NMT)

Number (n)

Short text message (SMS)

Third generation mobile phones (3G)

Watt (w)

## Competing interests

The author(s) declare that they have no competing interests.

## Authors' contributions

FS was the principal investigator responsible for design, conduct, analysis, interpretation of data and writing the manuscript.

LH made contributions to conception and design and also to analysis and drafting the manuscript.

MC participated as statistician and in the compilation and interpretation of the data for this publication.

KHM gave advice on the compilation of data for this publication.

## Pre-publication history

The pre-publication history for this paper can be accessed here:


